# The impact of novel anchored barbed suture for capsular closure on hospital length of stay after total knee arthroplasty: a retrospective cohort study

**DOI:** 10.1186/s12891-022-05292-y

**Published:** 2022-04-11

**Authors:** Liming Zhao, Wen Su, Zheyu Huang, Zhimin Zeng, Zhenglin Di, Kun Tao

**Affiliations:** 1 Department of Orthopedics, Ningbo The Sixth Hospital, No. 1059 East Zhongshan Road, Ningbo, Zhejiang, 315040 China; 2 Health Economic Research Institute, School of Pharmaceutical Sciences, Sun Yat-sen University, Guangzhou, China

**Keywords:** Barded suture, Capsular closure, Length of stay, Enhanced recovery after surgery (ERAS)

## Abstract

**Objective:**

The aim was to evaluate whether using novel anchored barded suture for capsular closure can further shorten the length of stay following primary total knee arthroplasty (TKA) within existed enhanced recovery after surgery (ERAS) protocol in osteoarthritis patients.

**Methods:**

A retrospective cohort study was conducted among osteoarthritis patients aged 18 to 80 years without major comorbidities who underwent primary unilateral TKA between January 2018 and December 2019 was conducted. The capsular closure techniques, interventions for ERAS, operation time and length of stay were collected via hospital electronic information system. Propensity-score matching was used to compensate for the difference in interventions for ERAS and patient characteristics. Subgroup comparison of patients treated under normal ERAS protocol was performed.

**Results:**

Included were 315 patients with capsular closure by barded suture and 397 patients with interrupted capsular closure by traditional suture. Patients’ characteristics and interventions for ERAS were balanced after propensity-score matching. The average postoperative length of stay in barded suture group was shorter than the compared group (2.10 ± 0.57 vs. 2.33 ± 0.80 days, *p* = 0.004), and with a significantly higher proportion of patients discharging within 2 days post procedure (88.0% vs. 70.7%, *p* < 0.001). The operation time for patients with barded suture closure was shorter compared to interrupted closure technique (100.90 ± 16.59 vs. 105.52 ± 18.47 min, *p* = 0.004). Subgroup analysis of patients treated under different levels ERAS protocol showed comparable results.

**Conclusion:**

The use of barded suture for capsular closure was associated with shorter length of stay after TKA compared to traditional suture, suggesting that barded suturing technique could be one effective intervention for ERAS.

**Supplementary Information:**

The online version contains supplementary material available at 10.1186/s12891-022-05292-y.

## Introduction

Knee osteoarthritis is the most common joint disorder in elderly individuals, which is a leading cause of global disability and is associated with significant economic costs as well as reduced quality of life [1, 2]. Total knee arthroplasty (TKA) is an effective treatment for end-stage knee osteoarthritis, and it has matured with decades of development and continued research [3–5]. The prevalence of symptomatic knee osteoarthritis in China was estimated to be around 8.1% in 2012 [6]. The prevalence has been growing significantly due to aging populations [7], and exerting increased constrains to limited medical resource. Therefore, it is important to establish practical strategies to achieve earlier recovery and better outcome for the patients undergoing TKA and at the same time, help reduce the heavy healthcare-related economic burden that comes with the increasing number of the procedures.

Enhanced recovery after surgery (ERAS) is defined as taking effective perioperative management therapies to reduce complications caused by surgery and improve the operation safety and patients’ quality of life. According to the ERAS Society, there are about 20 components of care that influence the stress response and enhance recovery [8]. These ERAS pathways have been reported to promote earlier recovery and be beneficial for patients [8–10]. In China, ERAS after TKA focuses on the improvement of surgical techniques and optimization of perioperative managements, including managing pain, reducing surgical blood loss, preventing infection and venous thromboembolism (VTE), and optimizing the use of drainage tube, catheter and tourniquet [11].

Novel anchored barbed sutures (barbed sutures) are often used in knee surgery to close the high-tension capsular layer. The STRATAFIX™ Symmetric PDS™ Plus Knotless Tissue Control Device comprises a portfolio of knotless barbed suture devices, which also have a triclosan coating to inhibit bacterial colonization and hence to reduce the risk of surgical-site infection. It is a more tensile strength barbed sutures which can provide superior tissue-holding capacity compared to traditional braided absorbable suture [12][{[Nawrocki, 2017 #2448]}]. STRATAFIX™ barbed suture can close the wounds substantially faster than using an interrupted technique, resulting in significant time saving in the OR [13]. There has been some published randomized controlled trials and observational studies which found that barbed sutures can reduce the closure time to enhance surgery efficiently while maintaining the same safety standard as traditional sutures [14–17]. However, there is still a lack of research on the using of different closing technologies in TKA to accelerate recovery after surgery and shorten the length of hospitalization in China.

The primary objective of the study is to evaluate whether barbed suture technology is a factor that influences the hospital length of stay in primary TKA patients in existing ERAS programs. The study is registered with Chinese Clinical Trial Register (ChiCTR2100043366).

## Methods

### Study design

**Fig. 1:**
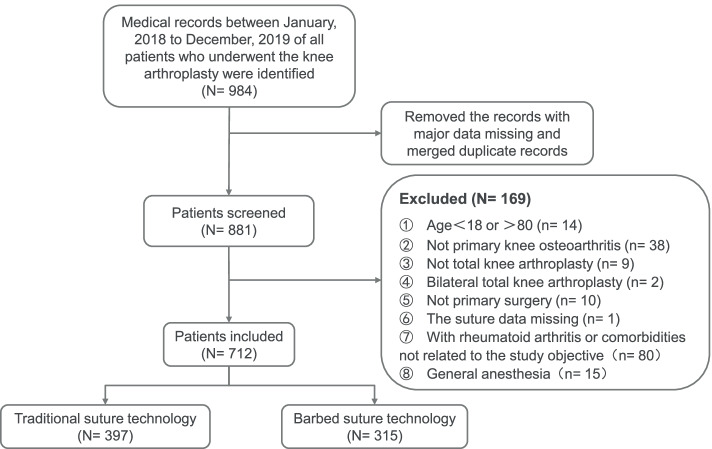
The flow of patients through the study

This single-center, retrospective cohort study used the de-identified data retrieved from the electronic medical records (EMR) at the Orthopedics Department at a tertiary orthopaedic specialized hospital in Ningbo, China, from January 2018 to December 2019. The study was approved by the Ethics Committee of Ningbo The Sixth Hospital (K2020009). The inclusion criteria were: (1) age 18 to 80 years old; (2) diagnosis of grade III or IV primary knee osteoarthritis; and (3) underwent primary unilateral total knee arthroplasty. Patients were excluded if they met any of the following criteria:(1) with comorbidities not related to the study objective or rheumatoid arthritis, which are listed in the Additional Table 1 ; (2) did not undergo spinal anesthesia; (3) had another surgery caused by incision during the hospitalization. Figure 1 illustrated the patient flow through the study.

### Closing Technique

All TKA procedures were conducted by the center orthopedic surgeon team in which all doctors have had at least 5 years’ experience. Continuous suture was used to close the joint capsule in all procedures and interrupted suture was used for skin closure. All patients used conventional absorbable suture for skin closure. Patients who used the barbed suture technology for joint capsule closure were allocated to barbed suture group, while the remaining patients who used the conventional suture were allocated to traditional suture group.

For the barbed suture group, the joint capsule was sutured with size 1 STRATAFIX™ Symmetric PDS™ Plus sutures. The sutures were introduced in the center of the wound and the surgeons ran the suture towards opposite ends of the wound simultaneously. For the traditional suture group, the joint capsule was sutured with absorbable size 1 bidirectional sutures which were placed from one end of the wound to the other side. Both groups used 2–0 absorbable suture subcutaneously, and 3–0 absorbable suture for the skin.

### Covariates

Patients’ demographic characteristic and medical history, ERAS programs, postoperative information were collected to set up the analytical database. The patient baseline characteristics that may affect the study outcomes of interest are considered in the statistical analysis. These factors included: age, gender, residence, BMI, smoking and drinking status, comorbidities, and medical conditions.

**Fig. 2:**
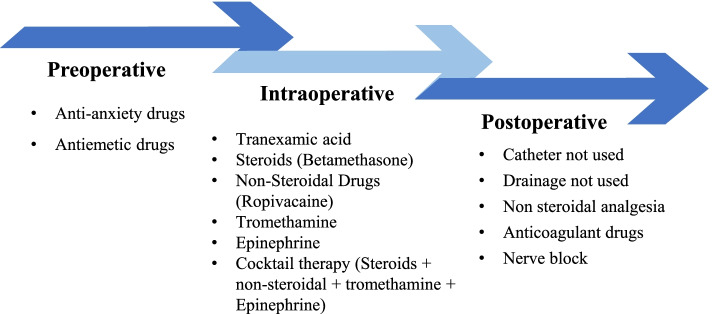
ERAS programs used in the center during 2018 and 2019

Over the time period from 2018 to 2019, there were thirteen ERAS programs being implemented in the center to enhance patient recovery. Of the 13 ERAS programs, anticoagulants were used routinely while other programs were used according to the patient’s actual situation. They included: tranexamic acid, four types of cocktail therapies (steroids, non-steroidal analgesia, tromethamine and adrenaline), catheter, drainage, non-steroidal analgesics, anxiolytics, antiemetics and nerve blocks. The ERAS programs information was identified from medical cost list in the EMR. The ERAS programs details are shown in Fig. 2 and the detailed extraction criteria are presented in the Additional Table 2.

There was a published nationwide observational study examined the relationship between the number of ERAS components implemented (‘level’) and perioperative outcomes [18]. And the result showed that the ERAS level of utilization was independently associated with incrementally reduced length of stay during the primary admission. So, this study identified three subgroups to explore the impact of barbed suture under different ERAS program levels. Among the abovementioned 13 ERAS programs, patients were categorized by the number of individual ERAS components used: ‘High ERAS’, ‘Medium ERAS’, and ‘Low ERAS’ level of use if they received either ≥8, 5–7, or ≤ 4 ERAS components, respectively.

### Statistical analysis

The study endpoints were the postoperative length of stay (LOS), discharge rate within 2 days after the index surgery, and operative time. The postoperative LOS was defined as the period between the index surgery date and first discharge date. Discharge rate within 2 days means the percentage of patients discharged within 2 days after the index surgery. Operative time was routinely recorded in the EMR system.

Descriptive analysis was reported as mean (standard deviation) for continuous variables, or count (percentage) for categorical variables. The Kruskal-Wallis test or t-test where appropriate for continuous variables and the Chi-Square test for categorical variables to determine whether the differences observed across the two groups were statistically significant. In situations where the sample size was insufficient to perform a Chi-Square test (fewer than 5 observations in a cell), the Fisher Exact test was performed. R (version 3.5.3) was used to perform the statistical analyses. The P values less than 0.05 were considered statistical significance (two-tailed test).

Propensity score matching (PSM) method [19] was employed to ensure balance of potential confounding factors between the two groups.

The propensity scores were generated by the logistic regression fitted to explain barbed and traditional suture techniques by independent variables: age, gender, residence, BMI, smoking and drinking status, comorbidities, medical conditions, tranexamic acid, four type of cocktail therapies (steroids, non-steroidal analgesia, tromethamine and adrenaline), catheter, drainage, non-steroidal analgesics, anxiolytics, antiemetics and nerve blocks.

Patients in the barbed suture group were matched to the traditional suture group using 1-to-1 nearest-neighbor matching technique without replacement and with a 0.01 caliper level.

Recovery after surgery was analyzed for subgroups stratified by various ERAS intervention levels, the subgroup was defined as high EARS, medium ERAS and low ERAS. All patients involved in this study were divided into these three subgroups according to the number of ERAS programs received. The differences in LOS, discharge rate within 2 days after the index surgery, and operative time by interventions were analyzed separately for the subgroups population.

## Results

### Baseline Characteristics

There was a total of 712 patients included, out of whom 315 patients received the barbed suture technology and 397 received the traditional suture technology. The two groups were comparable on demographic and clinical characteristics, including age, gender, residence, BMI, smoking and drinking status, comorbidities, and medical conditions (all *p* values >0.05) (Table 1). However, some ERAS programs had significant differences between the two groups, so that PSM analysis was used to adjust the potential differences. In the barbed suture group, patients had significantly higher rates (59.4% vs. 36.3%, *p* < 0.001) of cocktail therapy and of utilization of steroids (betamethasone), tromethamine, and epinephrine (all *p* < 0.001). In contrast, patients in traditional suture group were mainly managed with nerve blocks (65.2% vs. 40.0%, *p* < 0.001).

**Table 1:** Baseline characteristics

	Traditional Suture	Barbed Suture	***P*** value	Matched-TS^**a**^	Matched-BS^**b**^	***P*** value
**Sample size**	397	314	\	257	257	\
**Age (years)**	67.54 ± 6.39	67.99 ± 6.24	0.348	68.02 ± 6.18	67.93 ± 6.43	0.872
**Gender, N (%)**						
Male	110 (27.7)	74 (23.5)	0.234	72 (28.0)	57 (22.2)	0.154
**Residence, N (%)**						
Ningbo	281 (71.1)	223 (70.8)	0.467	179 (69.9)	181 (70.4)	0.846
Other citys in Zhejiang province	40 (10.1)	40 (12.7)		27 (10.5)	30 (11.7)	
Other provinces	74 (18.7)	52 (16.5)		50 (19.5)	46 (17.9)	
**BMI (kg/m2)**	26.02 ± 3.51	25.75 ± 3.48	0.548	25.86 ± 3.63	25.78 ± 3.53	0.872
**Smoking, N (%)**	4 (4.3)	11 (5.4)	1	3 (4.0)	9 (5.4)	0.759
**Drinking, N (%)**	12 (13.0)	18 (8.9)	0.373	11 (14.7)	13 (7.8)	0.159
**Comorbidities, N (%)**						
Diabetes	44 (11.1)	29 (9.2)	0.487	20 (7.8)	25 (9.7)	0.532
Hypertension	156 (39.3)	117 (37.1)	0.611	100 (38.9)	93 (36.2)	0.585
Hyperlipidemia	8 (2.0)	4 (1.3)	0.563	4 (1.6)	2 (0.8)	0.686
Hepatitis	9 (2.3)	4 (1.3)	0.405	7 (2.7)	3 (1.2)	0.339
Lumbar spine lesions	5 (1.3)	3 (1.0)	1	4 (1.6)	2 (0.8)	0.686
Heart diseases						
Coronary heart disease	7 (1.8)	7 (2.2)	0.868	6 (2.3)	6 (2.3)	1.000
Others	7 (1.8)	7 (2.2)	0.868	5 (1.9)	4 (1.6)	1.000
Digestive tract disease	6 (1.5)	6 (1.9)	0.911	4 (1.6)	4 (1.6)	1.000
Renal disease	4 (1.0)	5 (1.6)	0.519	4 (1.6)	4 (1.6)	1.000
Endocrine disease	6 (1.5)	2 (0.6)	0.312	4 (1.6)	1 (0.4)	0.373
Mental disease	3 (0.8)	3 (1.0)	1	2 (0.8)	2 (0.8)	1.000
**Preoperative hemoglobin < 100 g/L, N (%)**	2 (0.5)	0 (0.0)	0.506	2 (0.8)	0 (0.0)	0.499
**Procedure time, N (%)**						
A.M.	234 (58.9)	206 (65.4)	0.092	151 (58.8)	162 (63.0)	0.366
P.M.	163 (41.1)	109 (34.6)		106 (41.2)	95 (37.0)	
**Procedure day of week, N (%)**						
Monday to Thursday	329 (82.9)	267 (84.8)	0.564	48 (18.7)	41 (16.0)	0.484
Friday to Sunday	68 (17.1)	48 (15.2)		209 (81.3)	216 (84.0)	
**ERAS programs, N (%)**						
Tranexamic acid	389 (98.0)	304 (96.5)	0.327	249 (96.9)	246 (95.7)	0.64
Steroids (Betamethasone)	171 (43.1)	233 (74.0)	<0.001	157 (61.1)	176 (68.5)	0.096
Non-Steroidal Drugs (Ropivacaine)	397 (100.0)	315 (100.0)	1	257 (100.0)	257 (100.0)	1
Tromethamine	159 (40.1)	198 (62.9)	<0.001	148 (57.6)	146 (56.8)	0.929
Epinephrine	178 (44.8)	218 (69.2)	<0.001	157 (61.1)	164 (63.8)	0.585
Cocktail therapy	144 (36.3)	187 (59.4)	<0.001	135 (52.5)	135 (52.5)	1
Catheter not used	81 (20.4)	42 (13.3)	0.017	34 (13.2)	34 (13.2)	1
Drainage not used	19 (4.8)	1 (0.3)	<0.001	6 (2.3)	1 (0.4)	0.122
Non-steroidal analgesia drugs (Celecoxib or Etoricoxib)	383 (96.5)	285 (90.5)	0.002	246 (95.7)	246 (95.7)	1
Anti-anxiety drugs	145 (36.5)	99 (31.4)	0.179	92 (35.8)	77 (30.0)	0.189
Antiemetic drugs	276 (69.5)	265 (84.1)	<0.001	207 (80.5)	207 (80.5)	1
Nerve block	259 (65.2)	126 (40.0)	<0.001	124 (48.2)	124 (48.2)	1

^a^*Matched-TS* Matched-traditional suture, ^b^*Matched-BS* Matched-barbed suture

### PSM analysis

The analysis included total 514 patients after PSM; 257 patients used the barbed suture in TKA and others used the traditional suture. There were no statistically significant differences in all ERAS programs characteristics between the two groups (Table 1). The barbed suture group was associated with significantly shorter operative time, compared with the traditional suture group (99.88 ± 14.89 vs. 104.50 ± 18.80 min, *P* = 0.016). For postoperative outcomes, the mean postoperative LOS was significantly shorter in the barbed suture group (2.10 ± 0.57vs. 2.33 ± 0.80 days, *P* = 0.004). Furthermore, the rate of discharge within 2 days after the index surgery was significantly higher in the barbed suture group (87.7% vs. 74.2%, *P* = 0.004) (Table 2).

**Table 2:** The clinical outcomes after PSM

	Traditional Suture(*n* = 257)	Barbed Suture(*n* = 257)	***P*** value
Operative time (minutes)	104.50 ± 18.80	99.88 ± 14.89	0.016
Postoperative length of stay (days)	2.33 ± 0.80	2.10 ± 0.57	0.004
Discharge within 2 days, N (%)	112 (74.2)	142 (87.7)	0.004

### Different levels ERAS groups analysis

For the total 712 patients, there were 438 patients received high level ERAS, 256 patients received medium level EARS and others received low level ERAS. The subgroup results were similar to PSM analysis, the results were presented in Table 3. In the medium ERAS group, the barbed suture group was associated with significantly shorter operative time and postoperative LOS compared with the traditional suture group (both *p* < 0.001). The rate of patients discharged within 2 days after the index surgery was also significantly higher in the barbed suture group (92.6% vs. 55.9%, *P* < 0.001). Even with the high-level ERAS programs, the rate of patients discharged within 2 days after the index surgery was still significantly higher in the barbed suture group (87.3% vs. 77.2%, *P* = 0.008). Meanwhile the postoperative LOS had no significant difference. For low ERAS group patients, there were only 2 patients used the barbed suture in TKA and 16 patients used the traditional suture. The operative time, postoperative LOS in the barbed suture group was shorter, and the rate of patients discharged within 2 days after the index surgery was higher. But the sample size was too small for meaningful statistical testing.

**Table 3:** The clinical outcomes of subgroup analysis

	Traditional Suture	Barbed Suture	***P*** value
***High ERAS***	*n* = 193	*n* = 245	\
Operative time (minutes)	102.06 ± 17.54	99.06 ± 16.46	0.066
Postoperative length of stay (days)	2.27 ± 0.71	2.15 ± 0.77	0.100
Discharge within 2 days, N (%)	149 (77.2)	214 (87.3)	0.008
***Medium ERAS***	*n* = 188	*n* = 68	\
Operative time (minutes)	110.63 ± 19.11	101.24 ± 13.69	<0.001
Postoperative length of stay (days)	2.58 ± 0.84	2.09 ± 0.33	<0.001
Discharge within 2 days, N (%)	105 (55.9)	63 (92.6)	<0.001
***Low ERAS***	*n* = 16	*n* = 2	\
Operative time (minutes)	133.75 ± 23.63	105.00 ± 21.21	0.122
Postoperative length of stay (days)	4.12 ± 1.71	2.00 ± 0.00	0.106
Discharge within 2 days, N (%)	2 (12.5)	2 (100)	0.057

## Discussion

To our knowledge, this is the first retrospective cohort study to investigate the impact of using different capsular closure techniques during TKA on hospital length of stay in China. With the ERAS programs being widely used, most of hospitals started to use multiple interventions to enhance recovery after TKA. These interventions may confound the outcomes when evaluating the effect of barbed suture technology. In this study, PSM and subgroup analysis were used to adjust the effects of these potential confounders. The difference is that the PSM method is based on the propensity scores of the two groups with a common value range (i.e., matched pairs had the similar probability of receiving barbed suture technology), while the subgroup analysis in this study is based on the number of patients received ERAS programs to homogenize the characteristics between two groups. The PSM results demonstrated that the average operative time and average LOS of patients with barbed suture technology were significantly shorter and the proportion of patients discharged from hospital within 2 days post-procedure was significantly increased. The medium ERAS subgroup analysis showed the same results with the PSM. In the high ERAS subgroup, the proportion of patients discharged from hospital within 2 days post-procedure of patients with barbed suture technology was still significantly increased. The non-statistical differences observed in low ERAS subgroup was explained by the low statistical power as a result of the small sample size. As an observational study, this finding also indicated that the ERAS has been widely used in China recently to improve perioperative outcomes in patients receiving TKA procedures.

Previous published studies showed that arthrotomy and subcutaneous closure time were significantly shorter with barbed sutures in TKA [15–17, 20]. The latest meta-analysis [21] identified 1472 TKAs (1270 patients) assessed in 13 randomized-controlled trials, which revealed that compared with traditional closure techniques, barbed sutures resulted in shorter total wound closure time (MD = -317.55, 95% CI [− 409.07–226.03], *p* < 0.001). It was similar to the results of this study, which exhibited that operative time among patients with barbed suture technology. There is a lack of research on the LOS in patients who used different suture technologies in TKA. The present study showed that compared with traditional suture technology, barbed sutures resulted in significantly shorter LOS and higher proportion of patients discharged within 2 days post-procedure. Findings from a previous randomized controlled trial showed that the barbed suture had significant less wound dehiscence and positive leak tests by providing a more watertight arthrotomy closure and more robust to cyclical loading [17]. As the leakage could increase the risk of periprosthetic infection significantly [22], the beneficial results on LOS found in this study is likely to be attributable to the less arthrotomy leakage with barbed suturing. Moreover, traditional suture requires multiple knots increasing the chance of stitch abscess formation and wound infection [23], which also could lead to the longer LOS.

The shortening of LOS also improves the efficiency of surgical operations in medical institutions. We analyzed the LOS of all patients who underwent primary TKA in the center during 2018 to 2019 based on the analytical database in this study, the result showed the average LOS was 7.14 days. The center currently has 20 beds in total and it is estimated that 800 operations would be performed annually. The results showed that compared with the traditional suture technology, the average LOS of patients with barbed suture technology was shortened by 0.23 days. Therefore, it is projected that additional 54 operations could be performed annually with the use of barbed suture.

The results of this study should be interpreted in the context of limitation of study design. As the study was a retrospective review of EMR, the findings could be distorted because of presence of selection bias. Even though all observed potential confounding variables were comparable between the two groups with PSM, it was still possible that some unobserved factors associated with study outcomes were imbalanced between the two groups. All patients in this study were from a single center and the findings may not be generalizable in other clinical settings. Nevertheless, the results are encouraging and confirmation of benefits of the barbed suture technology requires further evaluation, preferably in a prospective, randomized setting.

## Conclusion

The novel anchored barbed suture for capsular closure was associated with a shorter hospital length of stay in patients receiving primary total knee arthroplasty. Even with the ERAS programs, the average operative time and average length of stay of patients with the novel anchored barbed suture were still significantly shorter.

## Supplementary Information

**Additional file 1.**

**Additional file 2.**

## Data Availability

The datasets generated and/or analysed during the current study are not publicly available because the data were from the Information Technology Department of Ningbo The Sixth Hospital, who applied restrictions on the data’s public availability. The datasets however are available from the corresponding author on reasonable request and with permission of the Information Technology Department.
