# Supplementary material for: The impact of novel anchored barbed suture for capsular closure on hospital length of stay after total knee arthroplasty: a retrospective cohort study
Source: BMC Musculoskelet Disord. 2022 Apr 11;23:349. doi: 10.1186/s12891-022-05292-y (PMC8996641; doi:10.1186/s12891-022-05292-y)
Supplement: Supplementary file 1 — Additional file 1. [file 12891_2022_5292_MOESM1_ESM.docx]

Additional table 1. Comorbidities not related to the study objective

| Disease name |
| --- |
| Secondary unilateral knee joint disease |
| Secondary bilateral knee joint disease |
| Gout |
| Gouty arthritis |
| Artificial hip joint disease |
| Hip joint disease |
| Hip replacement |
| Rheumatism |
| Type 2 diabetic peripheral neuropathy |
| Type 2 diabetic peripheral vascular disease |
| Type 2 diabetes with multiple complications |
| Type 2 diabetic nephropathy |
| Type 2 diabetic retinopathy |
| Rheumatoid arthritis |
| Ankylosing spondylitis |
| Infectious arthritis |
| Systemic osteoarthritis |
| Primary knee joint disease |
| Septic arthritis |
| Aseptic necrosis of femoral head |
| Osteoporosis with pathological fracture |
| Thoracic spine fracture |
| Lumbar fractures |
| Popliteal artery injury |
| Acute cerebrovascular disease |
| Acute bronchitis |
| Chronic renal failure |
| Polyarthritis |
| Primary bilateral hip joint disease |
| Vertebrobasilar Syndrome |
| Lung infection |
|  |
| Disease name |
| Acute upper respiratory tract infection |
| Chronic bronchitis |
| Urinary tract infection |
| Anemia |
| Hypoproteinemia |
| Mild anemia |
| Atrial fibrillation |
| Coronary artery stent implantation state |
| Pacemaker implantation NOS |
| Pacemaker installation |
| Has pacemaker |
| Radiofrequency ablation |
| Ventricular self-pulsation |
| Cerebral infarction |
| Sequelae of cerebral infarction |
| Lacunar infarction |
| Cerebral embolism |
| Angina pectoris |
| Arrhythmia |
| Ventricular hypertrophy |
| Aortic calcification |
| Left anterior branch block |
| Posterior circulation ischemia |
| Abnormal heartbeat |
| Parkinson |
| Alzheimer |
| Sequelae of polio |
| Psora |
| Old femoral neck fracture |
| Sjogren's syndrome |
| Connective tissue disease |
| Inferior vena cava thrombosis |
