# Supplementary material for: The impact of novel anchored barbed suture for capsular closure on hospital length of stay after total knee arthroplasty: a retrospective cohort study
Source: BMC Musculoskelet Disord. 2022 Apr 11;23:349. doi: 10.1186/s12891-022-05292-y (PMC8996641; doi:10.1186/s12891-022-05292-y)
Supplement: Supplementary file 2 — Additional file 2. [file 12891_2022_5292_MOESM2_ESM.docx]

Additional table 2. ERAS programs identification in the database

| Period | ERAS intervention | Data source | Detail |
| --- | --- | --- | --- |
| Intraoperation | Tranexamic acid | Fee | item name includes 'Tranexamic acid' |
| Postoperation | Cocktail therapy  (steroids) | Fee | item name includes 'Betamethasone' and type is 'injection' |
| Postoperation | Cocktail therapy  (non-steroidal analgesia) | Fee | item name includes 'Tromethamine' and type is 'injection' |
| Postoperation | Cocktail therapy  (tromethamine) | Fee | item name includes 'Ropivacaine' and type is 'injection' |
| Postoperation | Cocktail therapy  (adrenaline) | Fee | item name includes 'Epinephrine' and type is 'injection' |
| Postoperation | Cocktail therapy (all) | Fee | all of above cocktail therapy |
| Postoperation | Catheter | Fee | item name includes 'Catheter' |
| Postoperation | Drainage | Fee | item name includes 'Drainage' |
| Postoperation | Non-steroidal analgesia drugs | Fee | item name includes 'Celecoxib' or 'Etoricoxib', and the quantity is greater than 2 |
| Preoperation | Anti-anxiety drugs | Fee | item name includes 'Diazepam', 'Haloperidol' or 'Zolpidem tartrate', and the quantity is at least 1 |
| Preoperation | Antiemetic drugs | Fee | item name includes 'Mosapride', 'Metoclopramide' or 'Ondansetron', and the quantity is at least 1 |
| Postoperation | anticoagulant drugs for preventing VTE | Fee | item name includes 'Rivaroxaban' or 'Apixaban' |
| Postoperation | Anticoagulant drugs | Fee | item name includes 'Low molecular heparin' |
| Postoperation | Nerve block | Fee | item name includes 'Nerve block' |
